# Supplementary material for: Rapid and Local Self-Healing Ability of Polyurethane Nanocomposites Using Photothermal Polydopamine-Coated Graphene Oxide Triggered by Near-Infrared Laser
Source: Polymers (Basel). 2021 Apr 14;13(8):1274. doi: 10.3390/polym13081274 (PMC8070893; doi:10.3390/polym13081274)
Supplement: Supplementary file 1 [file polymers-13-01274-s001.pdf]

polymers MDPI

Supplementary Materials

Rapid and Local Self-healing Ability of Polyurethane Nanocomposites using Photothermal Polydopamine-Coated Graphene Oxide Triggered by Near-Infrared Laser

Yu-Mi Ha [1,†], Young Nam Kim [1,†] and Yong Chae Jung [1,*]

[1] Institute of Advanced Composite Materials, Korea Institute of Science and Technology, 92 Chudong-ro, Bongdong-eup, Wanju-gun, Jeollabuk-Do 55324, Republic of Korea; yumiha87@gmail.com
† These authors contributed equally.
* Correspondence: ycjung@kist.re.kr; Tel.: +82 (63) 219-8153

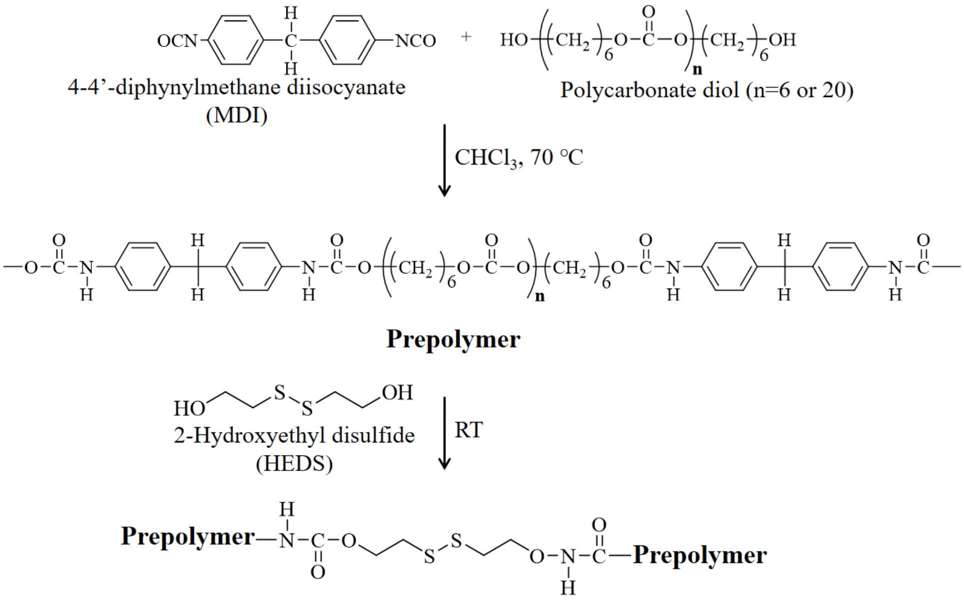

Scheme S1. Synthetic procedure of polyurethane with disulfide bonds.

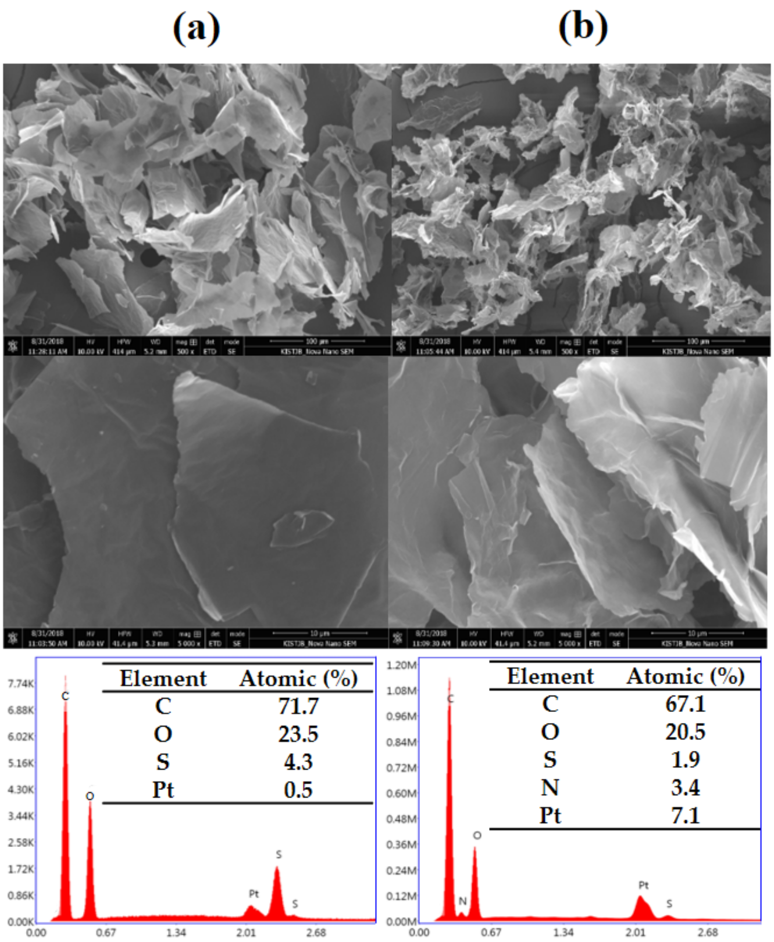

Figure S1. FE-SEM images and EDX of (a) GO, and (b) PDA-rGO powders.

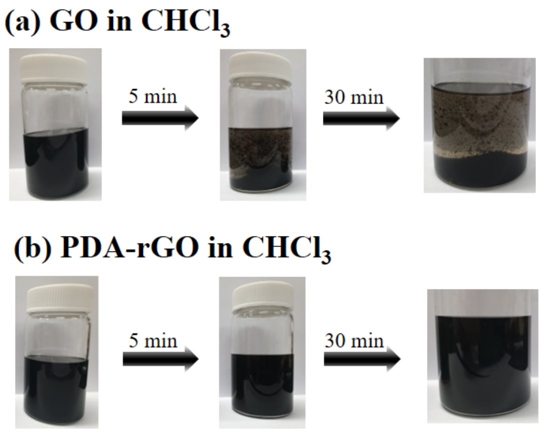

Figure S2. Photo images showing dispersion stability of (a) GO, and (b) PDA-rGO in chloroform.

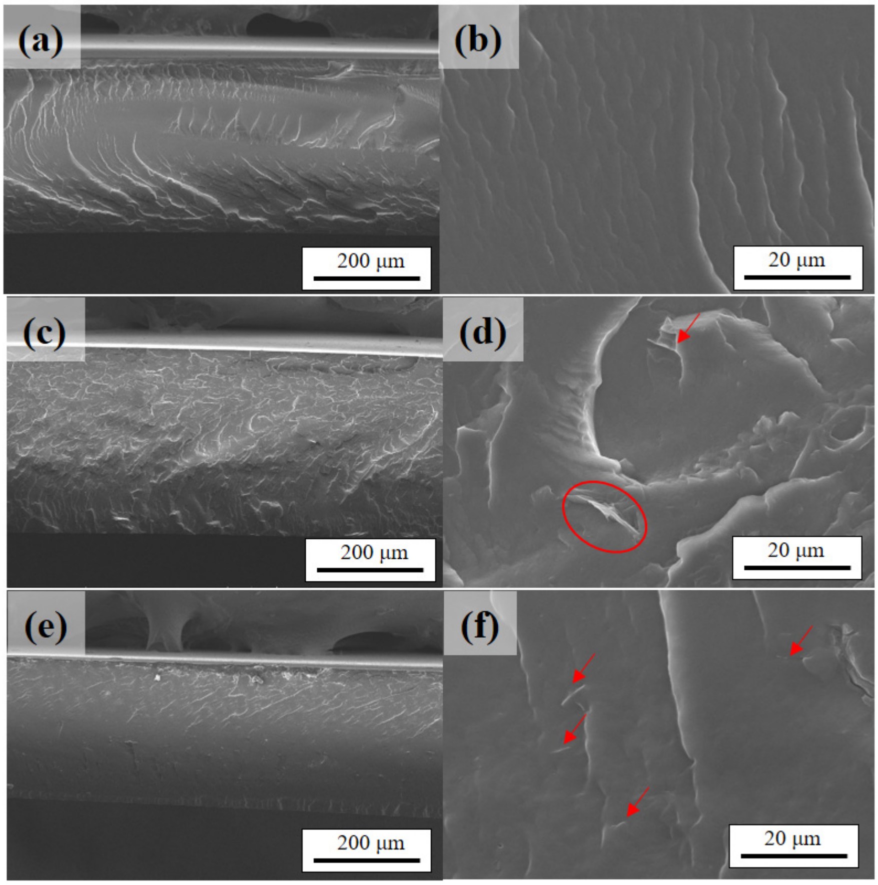

Figure S3. FE-SEM images of (a-b) PU, (c-d) 1 wt% GO/PU, and (e-f) 1 wt% PDA-rGO/PU nanocomposites.
